# Two Rare Cases of Long Surviving Riboflavin Transporter Deficiency with Co-Existing Adenosine Monophosphate Deaminase (AMP) Deficiency

**DOI:** 10.3390/brainsci12121605

**Published:** 2022-11-23

**Authors:** Lin Zhang, Dominic Thyagarajan

**Affiliations:** 1 Department of Neuroscience, Central Clinical School, Monash University, Melbourne, VIC 3004, Australia; 2 Department of Neuroscience, Eastern Health, VIC 3128, Australia; 3 Department of Neuroscience, The Alfred Health, Melbourne, VIC 3004, Australia

**Keywords:** riboflavin transporter deficiency, adenosine monophosphate deaminase, AMPD1 mutation

## Abstract

(1) Background: Riboflavin transporter deficiency (RTD), formerly known as Brown–Vialetto–Van Laere syndrome, is a rare condition that causes a progressive neurological syndrome in early life with features of auditory and optic neuropathy, weakness of bulbar muscles and the diaphragm and sensorimotor neuropathy. Pathologic mutations in the genes that code for riboflavin transporters have been identified as the genetic basis of RTD, and the majority of the genetically confirmed cases are caused by mutations of *SLC52A3,* a riboflavin transporter 2 coding gene or compound mutations in *SLC52A2*, encoding riboflavin transporter 3. Fatality in childhood is common if the condition is left untreated, but survival into adulthood has been reported in cases treated with high-dose oral riboflavin. (2) Case summary: We report two long-term survivors of RTD type 2 due to compound heterozygous 185T> G and 1258G>A mutations in gene *SLC2A2*. They are two brothers in a family in which two female siblings died in childhood from a similar neurological disorder. Brother one, the older RTD survivor, is aged 71, and brother two is aged 58. Both have significant visual impairment from optic nerve atrophy and sensory ataxia. Their muscle biopsies showed decreased muscle adenosine monophosphate (AMP) deaminase activity. No AMPD1 mutation was detected through whole-genome sequencing. (3) Conclusion: Co-existing riboflavin transporter deficiency (RTD) type 2 and muscle AMP deaminase deficiency has not been previously reported. Apart from the possibility that there is a milder phenotype associated with these mutations in *SLC2A2*, AMP deaminase deficiency might have contributed to a survival benefit by preserving muscle function through accumulating intracellular AMP.

## 1. Introduction

Riboflavin transporter deficiency (RTD), formerly known as Brown–Vialetto–Van Laere syndrome, is a rare autosomal recessive neurodegenerative condition in early life, characterised by cranial, often auditory and/or optic neuropathies, upper and lower motor neuron signs, and motor and sensory neuropathy, which can mimic amyotrophic lateral sclerosis or mitochondrial disease [1,2,3,4]. Axonal sensorimotor neuropathy is a frequent finding in nerve conduction studies, and axonal degeneration has been detected on sural nerve biopsy [5]. In the central nervous system, neuronal loss and gliosis have been found in cranial nerve nuclei, tracts, and the midbrain. Brain imaging is normal in most published cases [5].

Loss-of-function mutations in riboflavin transporter-encoding genes, *SLC52A3* and *SLC52A2*, have been found to be responsible for most genetically confirmed RTD cases, named RTD type 3 and type 2, respectively [5,6]. The lack of intracellular riboflavin in RTD, with or without a reduction in the plasma riboflavin level, has been postulated to cause mitochondrial dysfunction as the biologically active forms of riboflavin are crucial coenzymes in numerous mitochondrial metabolic pathways [7,8]. Fatality in untreated cases usually occurs in childhood, whereas treatment with high-dose oral riboflavin supplements improves clinical outcomes in most cases [5,9,10,11].

Adenosine monophosphate deaminase (AMPD) is an enzyme predominantly expressed in skeletal muscle. It catalyses the conversion of adenosine monophosphate (AMP) to inosine monophosphate (IMP) and ammonia in the purine nucleotide cycle, an important metabolic cycling process in the maintenance of appropriate cellular energy supply [12,13]. In high-energy-consuming states in the skeletal muscle, where oxygen supply is limited, there is fast ATP-ADP turnover (2ADP ↔ ATP + AMP), named the myokinase reaction [12]. As a result of this reaction, there is an accumulation of intracellular AMP, which can dephosphorylate to adenosine and diffuse to the extracellular space. Concurrently, the activity of AMP deaminase is increased to convert AMP to IMP [12]. IMP is subsequently reconverted to AMP via the action of adenylosuccinate synthetase [12,13]. As such, during and following a sprinting exercise, there is a temporary reduction in the total muscle adenine nucleotide pool (ADP, ATP, and AMP) but an elevation in the IMP level [14]. 

AMPD deficiency is a relatively common finding in muscle biopsies, with a reported prevalence of 1–2% in the general population [15]. Its clinical significance remains debatable. Some have thought that it causes exercise-induced myalgia and fatigue [16,17,18,19,20,21]. The inherited form is caused by a nonsense mutation of the AMPD1 gene, reportedly more prevalent in white and black ethnic groups [15]. An acquired form in the presence of established neuromuscular or muscular conditions has also been described [22].

We report two brothers, both rare long-term survivors of RTD type 2 with co-existing muscle AMPD deficiency. The index case, now aged 71, is, to our knowledge, the oldest survivor so far.

## 2. Case History

The index case was a 48-year-old male engineer who presented to a tertiary movement disorders clinic in 2000 for a second neurological opinion on symptoms indicative of mitochondrial disease. He reported a gradual onset of visual impairment from age 9, which had plateaued at the age of 12. He had been otherwise well until the development, in his early 30s, of a bilateral sensorineural hearing loss confirmed on audiometry, ataxic gait, and impaired fine motor movements of the hands whilst still maintaining good overall muscle strength and exercise capacity.

Neurological examination revealed atrophic optic discs, bilateral sensorineural hearing loss, and signs of sensory neuropathy (absent ankle reflexes, distal lower limb loss of proprioception, and loss of pinprick and temperature sensation in stocking distribution) with a sensory ataxic gait. Muscle strength was normal. There were no upper motor neuron signs.

His birth history and development in early life were normal. He had no other medical comorbidities except for renal calculi following a decade of excessive cow milk consumption of approximately 8 L per day.

The index case’s family history was significant for having three siblings affected by a similar syndrome. Two of the three female siblings were found to have poor vision before school age, and both died around age 9 after developing hearing loss, muscle wasting, and weakness. The index case’s younger brother had a much later onset of symptoms. He developed visual impairment at age 19, followed by sensory ataxia and mild muscle weakness. The elder sister of the index case was the only unaffected individual in his generation. Their parents were non-consanguineous. Neither was known to have neurological symptoms, although the index case’s mother died at age 43 without further available information. There was no history of seizures or cognitive impairment in the family.

## 3. Investigations and Results

In the presence of optic atrophy on ophthalmoscopy and peripheral sensorimotor neuropathy in the lower limbs, the index case and his brother underwent genetic testing for spinocerebellar atrophy and mitochondrial disease, with no mutation detected. Refsum’s disease was excluded by a normal phytanic acid level, and mutations in OPA1 mitochondrial dynamin-like GTPase were absent. 

The original nerve conduction studies are not available.

On the muscle biopsy, both cases had essentially normal stains for mitochondria with only a single ragged-red fibre and the indented COX-negative fibres noted in the proband muscle sample. Neither had large deletions or rearrangements of the mitochondrial genome on a Southern blot. Common mutations for MELAS and MERRF were not detected. Enzyme histochemistry showed normal random checkerboard staining with mild 2B fibre atrophy. Both brothers had markedly reduced AMPD staining. No inflammatory cells were seen. 

The index case had MRI brain scans in 2008 and 2016. Both showed bilateral optic nerve atrophy and non-specific periventricular deep white T2 hyperintensities. 

In 2016, two years after next-generation sequencing became available in Australia, the two brothers and their older sister underwent whole-genome sequencing to solve the diagnostic mystery. Both brothers were found to have compound heterozygous mutations in the *SLC2A2* gene, 185 T> G and 1258G> A, whereas their older sister does not carry any *SLC 2A2* genetic mutation. In 2018, a repeat whole-genome sequencing was performed. The above genetic variants were replicated, and a specific search for AMPD1 genetic mutations was negative in both patients. Thus, they appear to have secondary AMPD deficiency. 

## 4. Treatment and Outcome

High-dose riboflavin was commenced as soon as the diagnosis was made, which halted the further progression of visual impairment in the index case’s brother but not in the index case. He self-ceased treatment after 12 months due to a lack of benefits. Apart from the established deficits, both brothers have enjoyed otherwise good health. 

## 5. Discussion

The two brothers are rare long-term survivors of untreated RTD type 2. The median survival of untreated cases is reported to be 7.5 years from symptom onset [5,23], with respiratory failure being the main cause of death. Nevertheless, both brothers were in their 60s when the treatment was initiated. The favourable outcome in these two cases may be attributed to the following factors. Firstly, RTD type 2, in comparison to RTD type 3, seems to have less respiratory involvement [11]. Secondly, certain genotypes may confer milder phenotypes, although genotype–phenotype association analysis is probably limited by the small number of cases. Thirdly, a novel dietary factor may be relevant: the index case reported an unusually high cow milk intake, 8 litres per day, from his early 20s until the development of kidney stones in his 30s. Cow’s milk is a major dietary source of riboflavin, with a concentration between 0.60 and 3.42 mg per litre [24,25]. Provided that his history was reliable, the index case’s daily riboflavin intake was close to 16 mg, nearly ten times the average daily adult requirement of 1.3–1.6 mg [26]. It might have served as a treatment for RTD even though the therapeutic dose of riboflavin is usually higher, between 7 and 60 mg/Kg [9,10,11]. Finally, the co-existing AMP deaminase deficiency might exert a favourable metabolic effect on the cardiovascular system. In support of this, homozygous or heterozygous mutations in the AMP deaminase gene have been linked to improved survival in heart failure and ischaemic heart disease [27,28]. One potential mechanism is the action of adenosine, a potent vasodilator [29]. Ischaemia in the heart muscle induces the accumulation of AMP [30], which dephosphorylates to adenosine. This accumulation of AMP could be further enhanced by the lack of AMP deamination due to AMP deficiency. In addition, Rannoe et al.’s study of metabolic–chronotropic response indicated that subjects with AMPD had more efficient oxygen delivery in their cardiovascular system [31]. Despite this, questions remain as to whether a survival benefit applies in the acquired form of AMPD, as in our RTD cases, and whether acquired AMPD is a protective mechanism in neuromuscular conditions. These questions can be further investigated by a detailed examination of the association between AMPD and the severity of metabolic myopathies.

Our case is unique in the literature on RTD because of the longevity of the proband. In this case, report, we suggest co-existing acquired AMP deficiency may ameliorate the severity of RTD.

## Data Availability

Not applicable.
